# The inhibitory effects of carvacrol, nystatin, and their combination on oral candidiasis isolates

**DOI:** 10.1186/s13104-024-06767-y

**Published:** 2024-04-11

**Authors:** Seyed Saeed Hosseini Balef, Seyed Sedigheh Hosseini, Negar Asgari, Ahmad Sohrabi, Nazanin Mortazavi

**Affiliations:** 1 Dental Research Center, Golestan University of Medical Sciences, Gorgan, Iran; 2 Laboratory Sciences Research Center, Golestan University of Medical Sciences, Gorgan, Iran; 3 Department of Mycology and Parasitology, Faculty of Paramedicine, Golestan University of Medical Sciences, Gorgan, Iran; 4 Infectious Diseases Research Center, Golestan University of Medical Sciences, Gorgan, Iran; 5 Radinmehr Veterinary Laboratory, Radin Makian Azma Mehr Ltd, Gorgan, Iran; 6 Department of Oral and Maxillofacial Medicine, School of Dentistry, Golestan University of Medical Sciences, Gorgan, PO Box 4916953363, Iran

**Keywords:** Oral candidiasis, *Candida albicans*, Nystatin, Carvacrol, Microbial sensitivity tests

## Abstract

**Background:**

*Candida*, a common oral microbiota, can cause opportunistic fungal infections. With rising *Candida* infections and limited effective antifungals, new treatments are needed. This study investigates carvacrol essential oil’s effect on oral candidiasis, alone and with nystatin, compared to nystatin alone.

**Materials and methods:**

In this study, oral samples were collected from dental clinic patients, especially denture users. The presence of *Candida* was confirmed and cultured from these samples. Candidiasis was detected by observing *Candida* colonies. Drug sensitivity was tested on 100 positive samples. The minimum concentration of inhibition and lethality of each isolate was evaluated using nystatin and carvacrol. The results were compared using two-way analysis of variance. Finally, the minimum inhibitory concentration (MIC) of nystatin and carvacrol was calculated individually and in combination.

**Results:**

The present study found that *Candida albicans* and non-albicans species were equally prevalent. Carvacrol showed significant biological activity against all *Candida* species, with an average MTT of 50.01%. The average MIC value of carvacrol was 24.96 µg/ml, indicating its potential to inhibit *Candida* growth. The mean Minimum Fungicidal Concentration (MFC) value of carvacrol was 23.48 µg/ml, suggesting its effectiveness in killing the fungi.

**Conclusion:**

The study’s findings reveal that the MIC of carvacrol was significantly lower than that of nystatin and the combination of nystatin and carvacrol. This suggests that carvacrol holds potential as an effective herbal remedy for candidiasis.

## Introduction

Oral candidiasis, also known as thrush, is a common opportunistic infection that arises from the overgrowth of various *Candida* fungal species, predominantly *Candida albicans* [1]. This fungal infection, known as oral candidiasis, is notably prevalent among individuals with compromised immune systems, such as those living with human immunodeficiency virus (HIV). It manifests as white, creamy patches on the tongue and inner cheeks. These lesions can cause symptoms like redness, soreness, a cotton-like sensation in the mouth, altered taste, and discomfort or pain when eating or swallowing. Rubbing or scraping the lesions may lead to minor bleeding. While the infection typically targets the oral mucosa, it can spread to the oropharynx and esophagus in more severe cases. The condition arises from an excessive growth of Candida species within the oral cavity, often triggered by an imbalance in the oral microbiota or a weakened immune system [2]. Broad-spectrum antibiotics are often implicated in the increased growth of *C. albicans* due to their impact on the body’s natural microbial communities. These medications can disrupt the microbial equilibrium in areas like the mouth and gastrointestinal tract, paving the way for fungal overgrowth. A disturbed microbiota balance means less competition for *Candida*, enhancing its ability to proliferate and raising the risk of infection [3, 4]. Investigations have illuminated the role of gut microbiota in the onset of candidiasis. While *Candida* colonization in the gut is a common occurrence that may predispose individuals to systemic candidiasis, under certain circumstances, it may also confer beneficial effects to the host [5]. Recent studies have highlighted a prevalent deficiency of vitamin D among HIV patients, which is closely linked to an increased susceptibility to oral candidiasis. These findings underscore the importance of considering vitamin D supplementation as a potential strategy to improve oral health and protect against oral thrush in those living with HIV [6]. *C. albicans*, found in the oral cavity of 40–60% of the general population, is the most prevalent oral microorganism [7]. In isolates of oral candidiasis, *C. albicans* is the most prevalent species, accounting for 61.6% of cases. Other species of *Candida*, including *C. krusei*, *C. lusitaniae*, *C. dubliniensis*, *C. kefyr*, *C. parapsilosis*, *C. tropicalis*, *C. glabrata*, and *C. guilliermondii*, are less frequently observed [8]. When the natural immunity of the host is weakened, *C. albicans* has the potential to cause either localized or systemic infections [9]. Various risk factors are associated with oral candidiasis, including compromised salivary gland function, use of dentures, disruption of the oral mucosa, usage of certain medications, age-related vulnerabilities, hormonal changes, specific dietary habits and cancer. The treatment strategy for oral candidiasis often focuses on mitigating these contributing factors [10]. A variety of both topical and systemic treatments are presently accessible for managing oral candidiasis [11]. Recent research has highlighted the antifungal properties of Neem (Azadirachta indica) extracts, particularly against *C. albicans*. One study demonstrated that Neem leaf extracts were as effective as 3% sodium hypochlorite and more effective than 2% chlorhexidine in suppressing *C. albicans* growth. Additionally, a review of Neem’s extensive antimicrobial effects noted its efficacy in treating dental plaque, gingivitis, and related pain, as well as its activity against biofilm-forming bacteria and *C. albicans*, both in vitro and ex vivo [12]. These findings underscore the therapeutic potential of Neem extracts and emphasize the importance of further research into their application for oral candidiasis and other fungal conditions [13]. Patients who do not respond to topical treatments or who are at a high risk of systemic infections may find systemic antifungal therapies beneficial. Conversely, for uncomplicated cases of oral candidiasis, topical antifungal medications such as clotrimazole, miconazole, and nystatin are typically recommended as the initial treatment approach [14]. Nystatin, a well-known polyene antifungal agent, is commonly prescribed for oral thrush. It functions by binding to ergosterol, a crucial component of fungal cell membranes. This interaction compromises the membrane’s integrity, leading to the leakage of essential cellular contents, ultimately causing the death of the fungus. Renowned for its effectiveness, nystatin is a reliable choice for treating oral candidiasis and has a history of positive treatment results [15]. However, it’s worth mentioning that nystatin can lead to undesirable side effects such as diarrhea, nausea and vomiting, and abdominal discomfort [16]. Therefore, recent research is exploring the use of herbal compounds as a more effective treatment for oral candidiasis, aiming to reduce side effects, particularly for denture wearers. Carvacrol is an aromatic composition of vegetable oils and the main composition of the essential oil obtained from thyme, which has recently been used in studies for its antifungal properties [17]. Compared to the results obtained in the production and delivery of pharmaceuticals, the development rate of antifungal drugs has been very slow, especially before the 1980s. For this reason, antifungal drugs are much less and more limited than antibacterial drugs. The increasing drug treatment of fungi and the resulting increase in the dosage of drugs and then the side effects of the drugs cause important factors to be focused on basic agents such as medicinal plants with much less side effects [18]. The practice of utilizing medicinal plants, either independently or in conjunction with modern pharmaceuticals, has gained popularity for its therapeutic benefits and potential to mitigate drug side effects. Consequently, the combined application of antifungal medications and plant extracts could potentially inhibit or slow down the development of fungal resistance to antifungal treatments [19].

As a result, we chose to examine the impact of carvacrol on *Candida* yeasts under laboratory conditions. We then compared these findings with the effects of nystatin on yeast, as well as the combined effect of carvacrol and nystatin on oral candida isolates. The aim was to utilize these findings to potentially incorporate this product in the treatment of candidiasis.

## Materials and methods

### Sample segregation

This study is descriptive-analytical research. The Ethics Committee of Golestan University of Medical Sciences approved the research protocol (IR.GOUMS.REC.1399.324). Upon obtaining ethical approval, two samples were collected from each participant presenting with denture-related symptoms of candidiasis at the Department of Oral Diseases. These samples were obtained by swabbing the same area of the oral mucosa. The first swab was used to prepare a direct slide, confirming the presence of Candida in clinical specimens. The second swab was used for culturing *Candida* isolates, facilitating their growth and determining drug sensitivity at a concentration of 10^5 CFU/ml. Colony counts were conducted for each Sabouraud dextrose agar culture medium. Individuals with a colony count exceeding 50 CFU were identified as positive for candidiasis. Upon confirmation of oral candidiasis, the individual’s sample was included in the study and forwarded to the laboratory for further analysis.

### Phenotypic identification of yeast species using candida chrome agar

This study involves the examination of two groups of *Candida* (Group 1: *Candida albicans* and Group 2: other *Candida* species). Within each of these two groups, namely *C. albicans* and other species, 11 samples were assessed under varying concentrations across all three drug categories. The objective was to determine the minimum inhibitory concentration (MIC).

### Study groups

Three categories of agents (nystatin, carvacrol, and a combination of nystatin and carvacrol) were used in the present study. The carvacrol group employed carvacrol in its Active Pharmaceutical Ingredient (API) form, which has an advantage over pharmaceutical formulations as it validates the anti-candidal effect of this substance in its pure form. The experimental design included a control group (nystatin group) and a combination group (nystatin and carvacrol), which served as a comparative group.

### Determining the MIC of the drug

This experiment utilized the CLSI-M27A3 method, a standard procedure for yeast susceptibility testing. Initially, 100 µl of RPMI-1640 medium (Sigma Aldrich, Germany) was added to a 96-well microplate. Subsequently, carvacrol oil (Sigma Aldrich, Germany) was prepared with ethanol and nystatin in 10% dimethyl sulfoxide (DMSO) (Merck, Germany) at varying concentrations. Each solution was added to the first well and serially diluted, resulting in a concentration range from 0.03 to 256 µg/ml. Utilizing the colorimetric method, a single yeast colony was taken from Sabouraud dextrose agar (SDA) and a suspension with 10^5 yeast cells was prepared in tubes with sterile serum. The suspension had an optical transmittance of 90% at 550 nm, which corresponds to 10^6 cells per milliliter. Then, 500 µl of the 10^6 cell suspension were added to 4.5 ml of sterile serum in another tube to achieve a concentration of 10^5 cells/ml. Subsequently, 10 µl of the suspension from each *Candida* isolate and standard strain (positive control) were added to the microplate. Initially, 100 µl of RPMI-1640 medium were added to the microplate wells. Then, 100 µl of extract, oil, or drug were added to the first well of each row, in serial dilution, resulting in a concentration range from 0.03 to 256 µg/ml. They were diluted from the second to the ninth well in the same manner. The tenth well (positive control) contained yeast suspension and RPMI-1640 medium without drugs. The eleventh well (negative control) contained medium without yeast. Then, 10 µl of *C. albicans* yeast suspension were added to all wells. After incubating for 24 h at 30 degrees Celsius (30 °C), 10 µl were taken from each well and inoculated on SDA with chloramphenicol. After another 24 h, the colonies and CFU were counted. The lowest concentration that inhibited yeast growth was defined as the MIC, and its lower dose was referred to as the Sub-MIC.

### Determination of minimum fungicidal concentration (MFC)

After conducting the MIC test, the minimum fungicidal concentrations of carvacrol, nystatin, and their combination were determined. These were identified based on the first dilution at which a decrease in the number of viable *Candida* cells was observed.

### Drug susceptibility test (MTT) of candidiasis isolates

After a 24-hour incubation period at 37 °C for yeasts, 25 µl of MTT solution (with a final concentration of 5 mg/ml, dissolved in deionized water) was added to the microplate. The plate was then incubated for an additional 3 h at 37 °C. Following this, the plate was centrifuged and the supernatant was discarded. To dissolve the resulting formazan, 1000 µl of isopropanol (made from 95 ml of isopropanol and 5 ml of 1 normal hydrochloric acid) was added to the precipitate. The optical absorption of formazan was then measured using an enzyme-linked immunosorbent assay (ELISA) reader at a wavelength of 550 nm.

### Statistical analysis

Data were analyzed using SPSS. Both qualitative and quantitative variables were described using appropriate indicators. The Shapiro-Wilk test was used to assess data normality. Depending on the distribution, either independent tests and analysis of variance or the Mann-Whitney and Kruskal-Wallis tests were used for analysis. A significance level of 0.05 was adopted.

## Results

### Prevalence of species

**Fig. 1:**
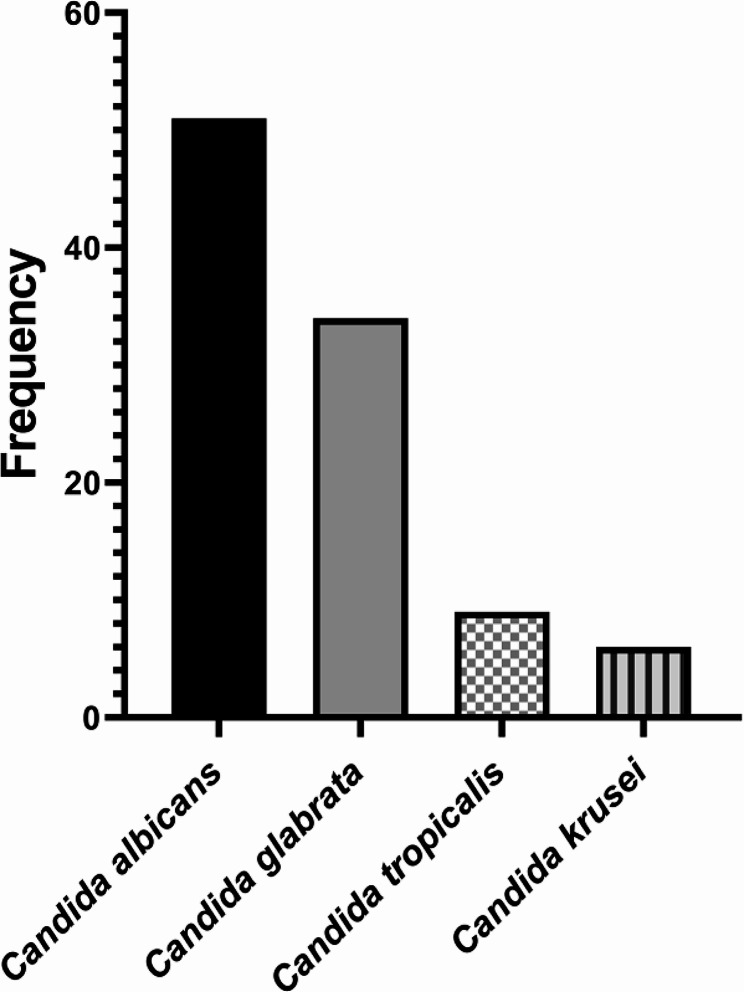
The percentage frequency of *Candida* species in oral samples from denture wearers. This bar graph shows the abundance of four different *Candida* species: *Candida albicans*, *Candida glabrata*, *Candida tropicalis*, and *Candida krusei*. As shown, *Candida albicans* has the highest frequency, close to 60, followed by *Candida glabrata* with a frequency around 40. Both *Candida tropicalis* and *Candida krusei* have a very low frequency, less than 10

In this study involving 100 denture-wearing patients, the prevalence of various *Candida* species was examined. *C. albicans* was found to be the most common, representing 51% of the cases. This was followed by *C. glabrata* at 34%, *C. tropicalis* at 9%, and *C. krusei* at 6% (Fig. 1).

**Table 1:** MIC, MFC and MTT test results

		MIC (µg/mL) Mean ± STD	MFC (µg/mL) Mean ± STD	MTT (%) Mean ± STD
***Candida albicans***	Carvacrol	22.87 ± 7.5	44.38 ± 14.29	49.97 ± 8.82
	Nystatin	81.67 ± 40.81	160.78 ± 82.35	30.75 ± 10.5
	Nystatin + Carvacrol	39.31 ± 21.17	80.56 ± 44.58	78.41 ± 11.34
	*p*-value	< 0.001	< 0.001	< 0.001
***Candida glabrata***	Carvacrol	27.30 ± 6.88	52.19 ± 13.77	50.2 ± 9.33
	Nystatin	80.24 ± 24.17	160 ± 49	31.2 ± 10.7
	Nystatin + Carvacrol	40.91 ± 22.25	82 ± 44	76 ± 10
	*p*-value	Not significant	Not significant	Not significant
***Candida krusei***	Carvacrol	28.71 ± 4.89	56.25 ± 8.79	43.93 ± 8.24
	Nystatin	75.83 ± 20.10	145 ± 43	35.3 ± 8.2
	Nystatin + Carvacrol	49.76 ± 16.81	99 ± 22	77 ± 10
	*p*-value	Not significant	Not significant	Not significant
***Candida tropicalis***	Carvacrol	25.47 ± 5.21	49.8 ± 10.77	53.51 ± 15.88
	Nystatin	74.78 ± 24.8	146 ± 45	29.6 ± 9.7
	Nystatin + Carvacrol	31.56 ± 13.49	63 ± 28	81 ± 10
	*p*-value	Not significant	Not significant	Not significant
***Candida (glabrata + krusei + tropicalis)***	Carvacrol	27.13 ± 6.36	52.25 ± 12.68	50.04 ± 10.76
	Nystatin	78.69 ± 23.49	155 ± 47	31.4 ± 10.2
	Nystatin + Carvacrol	40.27 ± 20.63	80 ± 41	77 ± 10
	*p*-value	< 0.001	< 0.001	< 0.001
**All species**	Carvacrol	24.96 ± 7.25	48.23 ± 14.03	50.01 ± 9.77
	Nystatin	80.21 ± 33.33	158 ± 67	31.1 ± 10.3
	Nystatin + Carvacrol	39.78 ± 20.81	81 ± 43	78 ± 11
	*p*-value	< 0.001	< 0.001	< 0.001

The Kolmogorov-Smirnov test results indicate a normal distribution for both the carvacrol group and the combination of nystatin and carvacrol in the MIC, MFC, and MTT tests.

Following the MIC test, the obtained values for all *Candida* species were as follows: 80.21 ± 33.33 µg/ml for nystatin, 24.96 ± 7.25 µg/ml for carvacrol, and 39.78 ± 20.81 µg/ml for the combination of nystatin and carvacrol. These averages were significantly different from each other (*p* < 0.05). The results indicate that carvacrol can effectively limit *Candida* growth at a minimum concentration of 24.96 µg/ml. Furthermore, when nystatin and carvacrol are combined, *Candida* species growth can be inhibited at much lower concentrations than when using nystatin alone. The MIC test results for *C. albicans* against carvacrol, nystatin, and their combination showed that minimum concentrations of 22.87 ± 7.50 µg/ml, 67.81 ± 81.40 µg/ml, and 17.21 ± 31.39 µg/ml, respectively could inhibit this species. The difference between the average MICs of the drugs was significant (*p* < 0.05), indicating that the MIC for each drug significantly differed from the others. The MIC test results for *C. glabrata*, *C. krusei*, and *C. tropicalis* against carvacrol were 27.3 ± 6.88 µg/ml, 71.28 ± 89.4 µg/ml, and 47.25 ± 21.5 µg/ml respectively. When compared to these species, the results using nystatin were 24.80 ± 17.24 µg/ml, 83.75 ± 10.20 µg/ml, and 78.74 ± 80.24 µg/ml, respectively. When nystatin was combined with carvacrol, the MIC for *C. glabrata*, *C. krusei*, and *C. tropicalis* were 91.40 ± 25.22 µg/ml, 67.49 ± 81.16 µg/ml, and 49.13 ± 56.31 µg/ml, respectively. Considering these three species as a non-albicans group due to their low frequency, the MIC test results for carvacrol, nystatin, and their combination showed that minimum concentrations of 27.13 ± 6.36 µg/ml, 78.69 ± 23.49 µg/ml, and 40.27 ± 20.63 µg/ml could inhibit this group respectively, with the differences in MICs being significant (*p* < 0.05).

The MTT test results showed that carvacrol had a significant inhibitory effect on the viability of all *Candida* species, with a mean value of 50.01 ± 7.79%. Nystatin had a lower effect, with a mean value of 10.31 ± 30.10%. The combination of carvacrol and nystatin had the lowest effect, with a mean value of 1.11 ± 0.78%. These differences were statistically significant (*p* < 0.05). For *C. albicans*, the mean MTT values for carvacrol, nystatin and the combination were 49.97 ± 8.82%, 75.30 ± 54.10% and 41.78 ± 34.11%, respectively. These differences were also statistically significant (*p* < 0.05), indicating that carvacrol had the highest inhibitory effect on this species. For non-*albicans Candida* species, including *C. glabrata*, *C. krusei* and *C. tropicalis*, the mean MTT values for carvacrol were 50.20 ± 9.33%, 43.93 ± 8.24% and 53.51 ± 15.88%, respectively. The mean MTT values for nystatin were 31.20 ± 10.70%, 35.30 ± 8.20% and 29.60 ± 9.70%, respectively. The mean MTT values for the combination were 76.00 ± 10.00%, 77.00 ± 10.00% and 81.00 ± 10.00%, respectively. These results showed that carvacrol had a higher inhibitory effect than nystatin on non-*albicans Candida* species, and the combination had a synergistic effect. The MTT test results confirmed that carvacrol had a potent antifungal activity against oral *Candida* isolates, and its combination with nystatin enhanced its efficacy.

In addition, the test results showed the antifungal effects of carvacrol, nystatin and their combination on different *Candida* species. Carvacrol is a natural compound found in some plants, while nystatin is a synthetic drug. In this experiment, the MFC test was used, which measures the lowest concentration of a substance that can kill the fungus.

This study showed that carvacrol has moderate antifungal activity against all *Candida* species with an MFC value of 23.48 ± 03.14 µg/ml. Nystatin was much more potent with an MFC value of 0.67 µg/ml. However, when carvacrol and nystatin were combined, their antifungal effect increased and the MFC value decreased to 00.81 ± 0.43 µg/ml. These differences were statistically significant (*p* < 0.05).

This study also compared the MFC values of the three materials for each *Candida* species separately. *C. albicans* was the most resistant to carvacrol (44.38 ± 14.29 µg/ml), but the most sensitive to nystatin (160.78 ± 82.35 µg/ml) and the combination (80.56 ± 44.58 µg/ml) was *C. glabrata*, *C. krusei* and *C. tropicalis* had similar MFC values for carvacrol (about 50 µg/ml), but differed in their response to nystatin and the combination. This paper categorized these three species as non-*albicans* and found that they had higher MFC values than *C. albicans* for all three substances (Table 1).

## Discussion

*Candida* is responsible for infections termed as candidiasis, which can manifest in various body parts such as the mouth, throat, intestines, and vagina. In severe instances, it can infiltrate the bloodstream and disseminate throughout the body, resulting in a potentially fatal condition known as invasive candidiasis. Hence, it is crucial to effectively treat *Candida* infections. Antifungal medications like nystatin are typically employed, but the rise in drug-resistant *Candida* strains necessitates the development of novel and potent treatments [20]. Resistance to nystatin in oral candidiasis develops due to several factors, including changes to ergosterol in the cell membranes of fungi, increased activity of efflux pumps that remove the antifungal agent, and biofilm creation that protects the fungal cells. These changes, coupled with genetic alterations, can greatly reduce the effectiveness of nystatin. Therefore, it’s essential to adapt treatment methods to address these resistance tactics, guaranteeing the successful control of this stubborn fungal condition [21, 22].

The antifungal properties of carvacrol stem from its ability to hinder the production of ergosterol and disrupt the structural integrity of the fungal cell membrane [23]. Research indicates that carvacrol poses a lesser risk than typical antifungal medications when compared to other substances for consumption [24]. By enhancing the anti-phospholipase activity, carvacrol hinders the specific functions of the fungal cell membrane. This not only curbs the microbial population but also increases the membrane permeability of fungi, rendering them susceptible to other antifungal agents [25]. Carvacrol boasts a broad spectrum of antimicrobial properties, which arise from its interaction with the microorganism’s cell membrane, leading to alterations in the permeability of ions such as potassium and hydrogen. Carvacrol, an isomer of thymol, shares a similar scent with it. This compound, derived from edible plants, is insoluble in water but soluble in alcohol and ether. It is present in the composition of edible plant oils like Origanum and in certain vegetable oils used as food flavorings [26, 27]. A study has examined the antifungal efficacy of carvacrol in conjunction with common antifungal medications such as nystatin and fluconazole for the treatment of *Malassezia pachydermatis* infections. The findings confirmed the synergistic properties of carvacrol [28]. Furthermore, the synergistic effect of carvacrol when combined with common antifungal medications in treating *C. auris* has also been explored and confirmed [29].

The current research establishes that *C. albicans* is the primary species of Candida found in individuals with dental prosthetics, a finding that echoes previous studies which have also recognized C. albicans as the dominant species in cases of oral candidiasis. Recent investigations support this conclusion, Qiu et al. (2023) [30] confirming the prevalence of *C. albicans* in both oral tissues and dental appliances. Additionally, the same study observed the presence of other *Candida* species such as *C. glabrata* and *C. tropicalis*, albeit to a lesser extent. We determined the MIC of the antifungal drug nystatin, the plant essential oil carvacrol, and a combination of the two against various *Candida* species. Our results indicated that carvacrol, particularly when combined with nystatin, significantly reduced the MIC against *Candida*, suggesting its potential as an effective, low-dose treatment option. In 2023, Powell et al. [31] reported that alginate oligosaccharides enhanced the antifungal activity of nystatin against *Candida* biofilms, leading to an up to 32-fold reduction in MIC. Additionally, the MTT test results indicated that carvacrol significantly inhibits the growth of *Candida* species, more effectively than nystatin. Carvacrol alone inhibited approximately 50% of *Candida*, while nystatin was less effective. When combined, they exhibited a synergistic effect, particularly against non-*albicans Candida*, suggesting the potential of carvacrol as an antifungal agent, especially when used alongside nystatin. Ismail et al. in 2022 [32] demonstrated that carvacrol inhibits the proliferation of *C. auris* at MICs ranging from 125 to 500 mg/mL.

In addition, our results indicated that the combination of carvacrol and nystatin is more effective against Candida, with a significant reduction in MFC observed in *C. albicans* compared to non-*albicans* species, which are less resistant to this combination.

A similar study by Mahboub and Tartor in 2020 [33] compared the antifungal effects of carvacrol and four other plant essential oils with several antifungal drugs. The essential oils demonstrated varying degrees of antifungal activity, with carvacrol showing the strongest effect against the fungi studied. Carvacrol also enhanced all immunological parameters. The study suggested that carvacrol could bilaterally inhibit fungal growth, making it a promising candidate for both external and internal antifungal treatments. In 2019, Schlemmer et al. [28] investigated the in vitro activity of carvacrol, cinnamaldehyde, and thymol in combination with antifungal drugs (fluconazole, itraconazole, ketoconazole, clotrimazole, miconazole, terbinafine, and nystatin) against the fungus *Malassezia pachydermis*. The most significant synergistic effects were observed when nystatin was combined with carvacrol or thymol, and when miconazole was combined with carvacrol. In a similar research approach, a 2021 study by Miranda-Cadena et al. [34]. investigated the effectiveness of plant essential oils, specifically carvacrol, cinnamaldehyde, and thymol, against *Candida* biofilms. The study found that cinnamaldehyde could significantly reduce the adhesion process of biofilms. However, carvacrol and thymol were found to significantly decrease the mass and metabolic activities of *Candida* biofilms. These findings suggest that these plant essential oils could be considered for use in treatment strategies aimed at either prevention or treatment of *Candida* infections. In our investigation, we found no notable variance in the efficacy of *C. albicans* groups compared to other species, attributable to the inhibitory influence of medicinal groups. However, a separate study conducted by Vitali et al. [35] in 2021 aimed at devising a pharmaceutical composition of carvacrol. They incorporated carvacrol into chitosan nanoparticles, leading to an augmentation in the inhibitory effect of carvacrol on diverse *Candida species* in both planktonic and biofilm states. These nanoparticles demonstrated distinct activity against planktonic forms. Conversely, their impact on biofilm forms was species-dependent, particularly noticeable against *C. tropicalis* and *C. krusei* species. Our study’s findings indicate that carvacrol was successful in diminishing the necessary concentration of nystatin when used in conjunction. A study conducted by Shaban et al. [29] in 2020 aimed to explore the impact of antifungal medications such as nystatin when combined with monoterpene phenols like carvacrol on the standard strain of *C. albicans*. They measured the MIC of antifungal drugs when used in combination with carvacrol. The study confirmed the synergistic attribute of the carvacrol and antifungal drug combination, which aligns with the conclusions drawn from our research. In addition, our research has been undertaken to understand how carvacrol inhibits *Candida*. A study led by Ismail et al. [32] in 2022, for instance, sought to uncover the mechanism through which carvacrol inhibits the activity of *C. auris* species. Their findings revealed that carvacrol influences the gene expression of antioxidant enzymes in the aforementioned species.

## Conclusion

Our observations reveal that carvacrol has moderate antifungal activity against *Candida* species, but by employing a combination of nystatin and carvacrol, we can achieve their inhibitory goal with lower drug concentrations. This implies that the concurrent use of these two drugs could potentially mitigate unwanted side effects associated with higher concentrations. Considering the distinct anti-*Candida* inhibition mechanisms of nystatin and carvacrol, the simultaneous administration approach could prove beneficial in overcoming active or resistant species of *Candida* in future studies. This advantage could potentially expedite the onset of the medicinal effect and also shorten the duration required for treatment. Based on these findings, we are optimistic about the potential utility of the carvacrol compound in treating fungal infections. The encouraging results related to its antibacterial effect suggest that it could serve as a multi-effect substance against microorganisms in oral infections in individuals with immune deficiencies due to various factors such as aging and diseases.

This paper also suggested that different species of *Candida* have different levels of sensitivity to the tested substances.

## Data Availability

No datasets were generated or analysed during the current study.

## Abbreviations

API: Active Pharmaceutical Ingredient
CFU: Colony Forming Unit
DMSO: Dimethyl Sulfoxide
ELISA: Enzyme-Linked Immunosorbent Assay
HIV: Human Immunodeficiency Virus
MFC: Minimum Fungicidal Concentration
MIC: Minimum Inhibitory Concentration
MTT: 3-[4,5-Dimethylthiazol-2-yl]-2,5-Diphenyl Tetrazolium Bromide
RPMI: Roswell Park Memorial Institute
SDA: Sabouraud Dextrose Agar
SPSS: Statistical Package for the Social Sciences
